# Atypical Enteropathogenic *Escherichia coli* Infection and Prolonged Diarrhea in Children

**DOI:** 10.3201/eid1204.051112

**Published:** 2006-04

**Authors:** Rang N. Nguyen, Louise S. Taylor, Marija Tauschek, Roy M. Robins-Browne

**Affiliations:** * University of Melbourne, Parkville, Australia;; † Murdoch Children's Research Institute, Parkville, Victoria, Australia

**Keywords:** Diarrhea, Escherichia coli, Escherichia coli infections, enteropathogenic E. coli, gastroenteritis, research

## Abstract

Some clinical isolates of enteropathogenic *Escherichia coli* (EPEC) lack bundle-forming pili and are termed atypical EPEC. The aim of this study was to determine if atypical EPEC are pathogens by comparing the clinical features of patients infected with atypical EPEC with those of children infected with other causative agents of diarrhea. Fecal samples obtained from children attending the Royal Children's Hospital in Melbourne for investigation of diarrhea were examined for adenovirus, rotavirus, *Campylobacter* spp., *Salmonella* spp., protozoa, and pathogenic *E. coli*. Clinical data were obtained by using a standardized pro forma and analyzed separately. Patients infected with atypical EPEC experienced mild, nondehydrating, and noninflammatory diarrhea that was not particularly associated with fever, vomiting, or abdominal pain. However, the duration of diarrhea in patients infected with atypical EPEC was significantly longer than that caused by the other species or where no pathogens were identified. Infection with atypical EPEC is associated with prolonged diarrhea.

## Abstract 2

Infection of children with atypical EPEC is associated with prolonged diarrhea.

The varieties of *Escherichia coli* that cause diarrhea are classified into pathogenic groups (pathotypes) according to their virulence determinants (1,2). The specific nature of these virulence determinants imbues each pathotype with the capacity to cause clinical syndromes with distinctive epidemiologic and pathologic characteristics (2). For example, enterotoxigenic *E. coli* causes watery diarrhea in children in developing countries and in travelers to those countries, whereas enterohemorrhagic *E. coli* (EHEC) may cause hemorrhagic colitis and the hemolytic uremic syndrome because of the production of Shiga toxins. Enteropathogenic *E. coli* (EPEC) shares several key virulence determinants with the most common varieties of EHEC but does not produce Shiga toxins nor cause hemorrhagic colitis or hemolytic uremic syndrome. Instead, it causes nonspecific gastroenteritis, especially in children in developing countries (3,4). EPEC also differs from other pathotypes of *E. coli* in that it typically carries an EPEC adherence factor plasmid (pEAF). This plasmid encodes 1) bundle-forming pili (Bfp), which promote bacterial adherence to epithelial cells and are an essential virulence determinant (5), and 2) a transcriptional activator, Per, that upregulates genes within a chromosomal pathogenicity island, termed the locus for enterocyte effacement (6,7). This pathogenicity island encodes a number of essential virulence proteins, including the surface protein intimin (the product of the *eae* gene), which is required to produce the attaching-effacing lesions that are a key feature of EPEC-induced pathology. A subset of EPEC, known as atypical EPEC, do not carry pEAF and hence do not produce Bfp or Per (4). Accordingly, their role in disease is controversial. Recently, we and others investigated the causes of community-acquired gastroenteritis in Melbourne (8,9). Among the infectious agents that were sought in these studies was atypical EPEC, which emerged as the single most frequent pathogen in the study population (9).

To determine if atypical EPEC are also responsible for diarrhea in hospitalized children, we undertook a comprehensive microbiologic study of patients with diarrhea at the Royal Children's Hospital in Melbourne.

## Patients and Methods

### Patients

Patients were children with diarrhea attending the Royal Children's Hospital, Melbourne, between March 1 and August 31, 2003. They were considered for inclusion in the study when an obviously loose stool sample from a child <14 years of age was received at the Diagnostic Microbiology Laboratory for investigation. After their caregivers, attending physicians, and medical records had been consulted, patients were considered eligible for inclusion in the study if, during the current illness, they had passed >3 loose stools within a day or had experienced loose stools plus vomiting, abdominal pain, or rectal bleeding. Patients with chronic gastrointestinal disorders, such as inflammatory bowel or celiac disease, were excluded, as were those with cystic fibrosis, leukemia, and other immunosuppressive disorders. Repeat samples and samples from children who had received antimicrobial agents within the preceding week were also excluded.

Clinical data were obtained in accordance with a standardized pro forma and were analyzed before the results of the laboratory findings were known. Data collected included age; gender; date of onset of illness; symptoms and clinical signs, including characteristics of stools, abdominal pain, vomiting (number per day and duration), fever, abdominal tenderness, largest number of bowel movements in a 24-hour period preceding the sample collection, and extent of dehydration. Duration of diarrhea was estimated from the passage of the first loose stool to the patient's last appearance in the ward or 1 day after discharge. Patients with temperatures of >38°C, taken by tympanic thermometer, were considered febrile. Severity of illness was estimated by using the 20-point scale developed by Ruuska and Vesikari (10).

### Laboratory Methods

All stool specimens were the first specimen obtained from a patient on a hospital visit, and specimens were investigated within 4 hours of collection. Specimens were examined macroscopically for color and consistency and by light microscopy for leukocytes, erythrocytes, and parasitic forms (amebas, cysts, and ova) by using a saline-and-iodine wet preparation and a modified Ziehl-Neelsen stain for oocysts of *Cryptosporidium* spp (11). Samples were tested by enzyme immunoassay for enteric adenoviruses and rotaviruses and cultured for *E. coli*, *Salmonella*, *Shigella*, *Yersinia*, and *Campylobacter* spp (12).

To reduce the cost of the investigation, diarrheogenic strains of *E. coli* were sought only during the first 11 weeks of the study, from March 1 to May 15. Bacteria were isolated from fecal samples by direct plating on MacConkey agar (Oxoid Ltd., Basingstoke, UK). After overnight incubation at 37°C, a sterile cotton swab was used to transfer the entire growth from each plate into Luria broth containing 30% (vol/vol) glycerol, which was then frozen at –70°C until required. *E. coli* pathotypes were identified by polymerase chain reaction (PCR) and confirmed by Southern hybridization (9). Briefly, template DNA for use in PCR was prepared from bacteria isolated from MacConkey agar plates and grown in 2.5 mL MacConkey broth with shaking at 37°C overnight. Bacteria from this culture were washed in phosphate-buffered saline, resuspended in sterile distilled water, and heated for 10 min at 100°C. Samples were then placed on ice for 5 min and recentrifuged for 5 min at 16,000 × *g*. Aliquots of the supernatant were pipetted into sterile tubes, stored at –20°C for <1 week, and then diluted 1 in 10 in distilled water before being added to the PCR mix. PCR amplifications were performed in a GeneAmp PCR System 9700 thermal cycler (Applied Biosystems, Foster City, CA, USA) with the PCR primers and conditions described previously (9). Genes identified by these primers and their association with each pathotype of diarrheogenic *E. coli* are listed in Table 1. PCR for the *lacZ* gene, which is present in almost all wildtype strains of *E. coli*, was included as a control to ensure that negative PCR results were not due to the absence of viable bacteria in the sample or the presence of inhibitors in the reaction mixture. Samples that were PCR negative for *lacZ* were excluded from further analysis. At the conclusion of the PCR, 10 μL of the reaction mixture underwent electrophoresis on 2.5% 96-well format agarose gels (Electro-fast; Abgene, Epsom, UK). Gels were stained with ethidium bromide, visualized on a UV transilluminator, and photographed. A portion of the PCR product was retained for Southern blotting, which was performed by using capillary transfer of separated DNA fragments onto positively charged nylon membranes (Roche Diagnostics Ltd., Lewes, UK). Digoxigenin-labeled DNA probes were prepared from the control strains of diarrheogenic *E. coli* listed in Table 1 and used as described (9). PCR- and probe-positive bacteria were assigned to a pathotype according to the criteria in Table 1. Equivocal or ambiguous assays were repeated, and if still unclear, were excluded from further analysis. Atypical EPEC strains were isolated in pure culture from the original sample and then serotyped by using hyperimmune rabbit antisera to O-antigens O1 through O181 (18). These strains were also subjected to PCR to determine the intimin subtype and to investigate the presence of selected virulence-associated genes by using the PCR primers and conditions described previously (9).

**Table 1:** Classification of pathogenic *Escherichia coli* according to amplicon(s) generated by polymerase chain reaction (PCR) for virulence-associated determinants

Interpretation†	Gene or virulence-associated determinant*										
	pCVD432	*aggA*	*bfpA*	*eae*	*ipaC*	*stIA*	*ltA*	*ehxA*	*stx1*	*stx2*	Control strain (reference)
EAEC‡	+	+	–	–	–	–	–	–	–	–	O42 (13)
Typical EPEC	–	–	+	+	–	–	–	–	–	–	E2348/69 (14)
Atypical EPEC	–	–	–	+	–	–	–	–	–	–	E128012 (14)
EIEC	–	–	–	–	+	–	–	–	–	–	223/83 (15)
ETEC‡	–	–	–	–	–	+	+	–	–	–	H10407 (16)
EHEC§	–	–	–	+	–	–	–	+	+	+	EDL933 (17)
STEC not EHEC¶	–	–	–	–	–	–	–	–	+	+	

*Factors specified by virulence genes: *aggA*, aggregative fimbria, AAF/I; *bfpA*, bundle-forming pilus; *eae*, intimin; *ipaC*, invasion plasmid antigen; *stIA*, heat-stable enterotoxin; *ltA*, heat-labile enterotoxin; *ehxA*, EHEC hemolysin; *stx*, Shiga toxin. †EAEC, enteroaggregative *E. coli*; EPEC, enteropathogenic *E. coli*; EIEC, enteroinvasive *E. coli*; ETEC, enterotoxigenic *E. coli*; STEC, Shiga toxin–producing *E. coli*; EHEC, enterohemorrhagic *E. coli*. ‡Either positive by polymerase chain reaction. §Either *eae* or *ehxA*, and either *stx1* or *stx2* positive. ¶Either s*tx1* or *stx2* positive.

### Statistical Analysis

Statistical analysis of quantitative and qualitative data was performed by using InStat, Version 3.05 (GraphPad Software Inc., San Diego, CA, USA). A 2-tailed p value of <0.05 indicated statistical significance. For the analysis of clinical features associated with infection, patients whose stools yielded >1 pathogen were excluded.

## Results

### Frequency of Viral, Parasitic, and Bacterial Pathogens

After exclusion of repeat samples and samples from patients >14 years of age or with cystic fibrosis, chronic inflammatory bowel disease, leukemia, or a history of recent antimicrobial drug usage, 303 of 972 consecutive fecal samples remained for analysis. Of these, 134 were from the first period of the study, March 1–May 15, when diarrheogenic *E. coli* were sought together with other enteropathogens, and 169 were from the period May 16–August 31, when *E. coli* were not sought.

The frequency of bacterial, viral, and parasitic pathogens identified during the 2 phases of the study are shown in Table 2. During the first period, a putative etiologic agent was identified in 88 (66%) of 134 children. Diarrheogenic *E. coli* were found in 42 (31%) of these children, followed by enteric adenovirus (10%), *Salmonella* sp. (10%), *Campylobacter* spp. (9%), *Giardia* sp. (6%), rotavirus (4%), and *Cryptosporidium* sp. (2%). Of the 42 *E. coli* isolates, 30 (71%) were EPEC; 6 (14%) were Shiga toxin–producing *E. coli* (STEC), of which 3 were EHEC; 4 (10%) were enteroaggregative *E. coli* (EAEC), 1 (2%) was enterotoxigenic *E. coli*; and 1 (2%) was enteroinvasive *E. coli*. Nine children (7%) were infected with >1 pathogen, including 2 concurrently infected with EPEC and adenovirus or EPEC and rotavirus, and 1 each with EPEC and *Giardia* sp.; STEC and *Campylobacter* sp.; STEC and *Giardia* sp.; EAEC and *Campylobacter* sp., and EAEC and rotavirus.

**Table 2:** Frequency of diarrhea-associated pathogens detected during the course of this study

Pathogen	1st period (Mar 1–May 15), n = 134 (%)	2nd period (May 16–Aug 31), n = 169 (%)
Diarrheogenic *Escherichia coli* (all)	42 (31.3)	Not done
*Campylobacte*r spp.	12 (9.0)	20 (11.8)
*Salmonella* spp.	14 (10.4)	9 (5.3)
Adenovirus	14 (10.4)	13 (7.7)
Rotavirus	5 (3.7)	57 (33.7)
*Giardia* sp.	8 (6.0)	3 (1.8)
*Cryptosporidium* sp.	2 (1.5)	1 (0.6)
>1 pathogen	9 (6.7)	4 (2.4)
No pathogens identified	46 (34.3)	70 (41.4)

All EPEC isolates were atypical EPEC (i.e., PCR negative for *bfpA*). Determination of the O:H serotype and intimin subtype of 29 of the 30 EPEC strains (1 was not viable) indicated that they were highly heterogeneous (Table 3). Although 3 strains (R41, R151, and R446) were O-nontypable:H34, intimin-α2; and 2 (R89 and R104) were O153:H7, intimin β, these isolates were neither temporally nor geographically related to each other and showed some differences in their carriage of accessory virulence-related factors (data not shown). Two other isolates (R250 and R436) were O33:H6 but had different intimin types. Ten isolates were O-serogroups that were classified as nontypable because they did not react with any of the available O-typing sera (O1–O181), and 2 isolates could not be serotyped because they were rough. Only 1 isolate (R404) belonged to an *E. coli* serotype, O128:H2, that is commonly associated with EPEC (4).

**Table 3:** Characteristics of atypical EPEC identified during this study*

Strain no.	Serotype	Intimin type
R41	ONT:H34	α2
R69	NT	θ
R89	O153:H7	β
R104	O153:H7	β
R151	ONT:H34	α2
R154	O71:H6	α1
R175	O128:H21	κ
R176	ONT:H8	ι
R182	OR:H40	θ
R215	O117:H2	ε/η
R218	ONT:R	α2
R219	O51:H49	α1
R227	O170/172:H49	θ
R228	O88:H-	κ
R249	O145:H34	ι
R250	O33:H6	ζ
R261	O2:H45	κ
R278	ONT:H19	υ
R281	O49:H10	κ
R380	ONT:H31	ζ
R392	ONT:H6	α2
R394	O5/71:H31	θ
R396	O98:H8	ι
R404	O128:H2	β
R420	ONT:H21	θ
R436	O33:H6	β
R446	ONT:H34	α2
R447	O28:H45	ζ
R457	ONT:H–	β

*EPEC, enteropathogenic *Escherichia coli*; H-, nonmotile; NT, nontypable (O1-O181; H1-H56); R, rough.

During the second period of the study, when *E. coli* was not sought, putative pathogens were identified in 99 (58.6%) of 169 children; rotavirus was the most frequent (33.7%), followed by *Campylobacter* (11.8%), adenovirus (7.7%), *Salmonella* (5.3%), *Giardia* (1.8%), and *Cryptosporidium* (0.6%) spp. Four patients were infected with >1 pathogen: 3 concurrently infected with rotavirus and adenovirus and 1 with adenovirus and *Salmonella* sp. *Shigella* and *Yersinia* spp. were not identified during either period of the study. The frequency of rotavirus infection during the second phase of the study was significantly greater than during the first phase (odds ratio [OR] 13.13; 95% confidence interval [CI] 5.08–33.91, p<10^–6^, 2-tailed Fisher exact test), confirming the well-known association of rotavirus with winter diarrhea (12). The frequency of patients in whom no pathogens were identified was not significantly different between the 2 study periods (OR 0.74, 95% CI 0.46–1.18, p>0.2, 2-tailed Fisher exact test), despite the omission of tests for diarrheogenic *E. coli* during the second period. This finding suggests that *E. coli* did not account for a large number of cases during the second period of the study and accords with our previous observations that diarrhea due to *E. coli* is relatively less frequent during winter (9,19).

### Comparison of Clinical and Laboratory Findings

The clinical and laboratory features of patients infected with different pathogens were compared (Table 4). Patients infected with >1 pathogen were excluded from this analysis, as were those infected with *Giardia* or *Cryptosporidium* spp. or diarrheogenic *E. coli* other than EPEC because their numbers were too small for the results to be meaningful. For this analysis, only those patients in whom no pathogens were identified from the first study period were considered because of the possibility that some of those studied during the second period were infected with diarrheogenic *E. coli*.

**Table 4:** Association between presumed etiologic agent and clinical and laboratory findings in children with diarrhea*

Clinical or laboratory parameter	Presumed etiologic agent					
	EPEC, n = 25	Adenovirus, n = 22	Rotavirus, n = 55	*Campylobacter,* n = 30	*Salmonella,* n = 22	NPI, n = 46
Age in months, median (interquartile range)	16.9 (11.4–28.2)	9.5 (4.4–19.5)†	15.2 (9.4–28.0)	34.2 (26.9–98.1)‡§	29.8 (9.3–90.9)¶	11.6 (3.1–52.7)
Sex, no. (% male)	18 (72)¶	14 (64)	30 (55)	17 (57)	10 (46)	20 (44)†
Vomiting, no. (%)	11 (44)	17 (77)†¶	49 (89)‡§	13 (43)	7 (32)	23 (50)
Abdominal pain, no. (%)	5 (20)	2 (9) ‡	10 (18)	17 (57)#**	10 (45)	10 (22)**
Days with diarrhea, mean (95% CI)	12.1 (7.5–16.7)¶	4.9 (3.6–6.2)‡	6.0 (4.9–7.1)‡	4.9 (3.9–5.9)‡	6.5 (4.2–8.6)†	6.3 (3.5–9.0)†
Diarrhea >14 days, no. (%)	12 (48)¶	1 (5)‡	3 (5)‡¶	1 (3)‡	2 (9)#	9 (20)†
Temperature >38°C, no. (%)	6 (24)	2 (9)	30 (55)†¶	7 (23)	17 (77)‡§	13 (28)
Dehydration >5%, no. (%)	1 (4)	2 (9)	35 (64)‡§	2 (7)	6 (27)†	6 (13)
Severity score, mean (95% CI)	9.0 (7.7–10.3)	8.6 (7.5–9.7)	13.5 (12.6–14.4)‡§	8.4 (7.5–9.3)	11.3 (9.8–12.8)#**	8.3 (7.4–9.2)
Fecal blood						
	Macroscopic, no. (%)	0	0	0	4 (13)	3 (14)	1 (2)
	Microscopic only, no. (%)	4 (16)	2 (9)	1 (2)	17 (57)¶§	9 (41)	9 (20)
	Fecal leukocytes, no. (%)	5 (20)	4 (18)	10 (18)	23 (77)‡§	14 (64)¶#	10 (22)

*EPEC, Enteropathogenic *Escherichia coli*; NPI, no pathogen identified; CI, confidence interval. †Significantly different from EPEC (Mann-Whitney U test, Fisher exact test, or Student *t* test, 2-tailed), p<0.05, >0.01. ‡Significantly different from EPEC (Mann-Whitney U test, Fisher exact test, or Student *t* test, 2-tailed), p<0.001. §Significantly different from NPI (Mann-Whitney U test, Fisher exact test, or Student *t* test, 2-tailed), p<0.001. ¶Significantly different from NPI (Mann-Whitney U test, Fisher exact test, or Student *t* test, 2-tailed), p<0.05, >0.01. #Significantly different from EPEC (Mann-Whitney U test, Fisher exact test, or Student *t* test, 2-tailed), p<0.01, >0.001. **Significantly different from NPI (Mann-Whitney U test, Fisher exact test, or Student *t* test, 2 tailed, p<0.01, >0.001.

Patients infected with EPEC were of a similar age (median 16.9 months) to those from whom no pathogens were isolated (median age 11.6 months). Of the various groups of patients defined according to the cause of diarrhea, only those infected with *Campylobacter* spp. (median age 34.2 months) differed significantly in age from those with EPEC (p = 0.0002, Mann-Whitney U test). Eighteen (72%) of 25 children monoinfected with EPEC were boys compared with 20 (44%) of 46 children in whom no pathogens were identified (OR = 3.34, 95% CI 1.17–9.55, p = 0.03, 2-tailed Fisher exact test), and with 49 (47%) of 104 children enrolled in phase 1 of the study who were not infected with EPEC (OR = 2.89, 95% CI 1.11–7.5, p = 0.03).

Patients infected with rotavirus or adenovirus were significantly more likely to have a history of vomiting than those with no pathogen identified or those infected with EPEC, *Salmonella*, or *Campylobacter* spp. The frequency of vomiting in patients infected with EPEC and in those with no pathogen identified was similar. Abdominal pain was reported significantly more frequently in patients infected with *Campylobacter* spp. than in those infected with EPEC (p = 0.007, 2-tailed Fisher exact test), adenovirus (p = 0.0005), rotavirus (p = 0.0005), or those in whom no pathogen was detected (p = 0.003).

The duration of diarrhea was significantly longer in patients with EPEC than in those infected with adenovirus (p = 0.002, 2-tailed Student *t* test), rotavirus (p = 0.0003), *Campylobacter* (p = 0.0003), *Salmonella* (p = 0.02), and those without an identifiable pathogen (p = 0.02). Moreover, persistent diarrhea (defined as diarrhea lasting >14 days) was significantly more common in patients infected with atypical EPEC than in those infected with adenovirus, rotavirus, *Campylobacter*, *Salmonella*, and those with no pathogen identified (Table 4). Persistent diarrhea also developed in 4 (36%) of 11 patients infected with *Giardia* sp. The frequency of persistent diarrhea associated with *Giardia* was significantly greater than that attributable to adenovirus (p = 0.03), rotavirus (p = 0.01), and *Camplyobacter*, but not *Salmonella* or atypical EPEC (p>0.1, 2-tailed Fisher exact test).

Fever was significantly more common in patients infected with rotavirus or *Salmonella* than in those infected with EPEC, adenovirus, or *Campylobacter*, and those with no pathogen identified. Dehydration of >5% occurred significantly more often in patients infected with rotavirus than in those infected with EPEC, adenovirus, *Campylobacter*, *Salmonella*, and those without an identifiable pathogen.

The disease severity score, determined according to the criteria of Ruuska and Vesikari (10), was highest in patients infected with rotavirus followed by *Salmonella* sp. The mean severity scores in patients infected with EPEC, adenovirus, and *Campylobacter* sp. and those in whom no pathogen was found were similar. Stools from patients infected with *Campylobacter* or *Salmonella* spp. were more likely to contain frank blood, although the differences between patients infected with different etiologic agents were not significant (p>0.05, 2-tailed Fisher exact test). Erythrocytes were more commonly detected on microscopic examination in patients infected with *Campylobacter* or *Salmonella* spp. than in those infected with EPEC, adenovirus, rotavirus, or no identifiable pathogen, but the differences were significant with respect to *Campylobacter* spp. only. Fecal leukocytes were present significantly more often in patients infected with *Campylobacter* or *Salmonella* spp. than in those infected with EPEC, adenovirus, rotavirus, or those with no identifiable pathogen.

## Discussion

The principal aims of this study were to compare the frequency of atypical EPEC with frequencies of established enteropathogens in children attending hospital with diarrhea and to determine the clinical and laboratory features associated with each pathogen. During the first part of the study (when pathogenic *E. coli* was sought), atypical EPEC was the predominant pathogen identified; it was found in 31% of 134 children compared with 10% for adenovirus, 10% for *Salmonella* sp., 9% for *Campylobacter* sp., and 4% for rotavirus. In the second period of the study, when EPEC was not sought, rotavirus predominated. In agreement with our findings from a community-based study in Melbourne and reports from investigators in Brazil, Norway, and elsewhere (9,20,21), the atypical EPEC strains obtained in this study were highly heterogeneous in terms of O:H serotype and intimin type, which indicates that the high frequency of atypical EPEC was not due to an outbreak caused by a limited number of strains. Also in agreement with our previous study, we observed that serotypes of EPEC associated with diarrhea differed from those listed by the World Health Organization as being characteristic of EPEC (9).

To determine whether atypical EPEC is a cause of diarrhea, we compared the clinical and laboratory findings of children who were infected with these bacteria with those who were infected with well-established pathogens and those in whom no pathogens were identified. The hypothesis underlying this investigation was that if atypical EPEC is not a pathogen, the symptoms, signs, and laboratory findings in patients infected with these bacteria would be the same as those in patients in whom no pathogens were found. The results showed that diarrhea attributable to atypical EPEC was significantly more common in boys and that it persisted significantly longer than diarrhea in patients without an identifiable pathogen or in those infected with adenovirus, rotavirus, *Campylobacter* spp., or *Salmonella* sp. This study also showed that infection with atypical EPEC generally occurred in children <2 years of age, with 72% <24 months of age compared with 55% for the first study group as a whole (OR 3.0, 95% CI 1.17–7.85, p = 0.03, 2-tailed Fisher exact test). Infection with EPEC was associated with vomiting in ≈50% of patients, was generally not accompanied by fever, abdominal pain, or dehydration, and was not characterized by fecal blood or leukocytes, indicating that it was not inflammatory in nature. The reason for the higher frequency of atypical EPEC in boys is not known but confirms our unpublished observations from a community-based study, in which 55 isolates were obtained from 338 male patients, and 34 were obtained from 358 female patients (OR 1.85, 95% CI 1.17–2.92, p = 0.009, 2-tailed Fisher exact test).

The validity of the clinical and laboratory assessments performed in this study was indicated by the confirmation of the well-known associations of specific pathogens with particular parameters: younger age of children infected with EPEC and viruses than those infected with *Campylobacter* or *Salmonella* spp.; rotavirus and *Salmonella* infections with fever; rotavirus with dehydration and an overall greater severity of disease; *Campylobacter* sp. with fecal blood; and *Campylobacter* and *Salmonella* spp. with fecal leukocytes (22).

Persistent diarrhea (lasting more then 14 days) eventually develops in a substantial proportion of children with acute infectious gastroenteritis and may become chronic, leading to malabsorption, failure to thrive, and malnutrition (23). A wide range of infectious agents has been implicated in the cause of persistent diarrhea, including viruses, in particular rotavirus; protozoa, such as *Giardia* and *Cryptosporidium* spp., and bacteria, including *E. coli* (23,24). In most cases, however, laboratory investigation of children with persistent diarrhea fails to yield an identifiable cause. The findings of this study suggest that a number of these cases may be caused by infection with atypical EPEC, which is seldom sought in these patients.

Despite the persuasive evidence of a volunteer study and reports of outbreaks of diarrhea attributed to atypical EPEC (25,26), the role of atypical EPEC in disease is controversial. In several reports, however, from countries as diverse as Iran, Norway, Peru, Poland, South Africa, the United States, and the United Kingdom (20,27–32), as well as Australia (9,33), atypical EPEC strains have been identified in children with acute diarrheal disease. Atypical EPEC has also previously been reported in association with prolonged diarrhea (34). For example, Hill et al. (35) reported that of 26 children infected with EPEC requiring hospital admission for acute diarrhea, life-threatening, chronic symptoms developed in 6 (23%). Five of these 6 children were infected with EPEC of serogroups O114 or O128, which frequently do not produce Bfp (14,36). Notwithstanding these previous reports, however, the current study is the first to characterize the illness caused by atypical EPEC in a systematic way and to compare the features of atypical EPEC infection with those of other etiologic agents of diarrhea.

The reasons why persistent diarrhea develops more frequently in children infected with atypical EPEC than in those infected with adenovirus, rotavirus, *Campylobacter* or *Salmonella* spp. are not known. In a recent study, Mellmann et al. (37) found that only 12 (<9%) of 137 patients who were infected with *eae*-positive EHEC strains when investigated within 14 days of the onset of diarrhea remained culture-positive when retested 3–16 days later, compared with all 5 patients who were initially infected with *eae*-positive, *stx*-negative *E. coli* (i.e., atypical EPEC) (OR 110.4, 95% CI 5.8–2117.6, p<0.0001, 2-tailed Fisher exact test). These findings indicate that atypical EPEC may have an innate propensity to persist longer in the intestine than varieties of *E. coli* which cause diarrhea that is more transient in nature. EPEC adheres tightly to epithelial cells and disrupts normal cellular processes (38), and evidence suggests that atypical EPEC may retard apoptosis of intestinal epithelial cells (39), possibly because of the lack of Bfp (40). These features may favor prolonged intestinal colonization by atypical EPEC compared with other intestinal pathogens. Although disease due to atypical EPEC was mild and generally not associated with dehydration, its importance lies in its association with prolonged diarrhea, a major contributor to childhood illness, especially in developing countries. Our findings also suggest that interventions targeted towards atypical EPEC may be beneficial in managing children with prolonged diarrhea.
